# Prognostic risk factors and nomogram construction for sebaceous carcinoma: A population-based analysis

**DOI:** 10.3389/fonc.2023.981111

**Published:** 2023-02-27

**Authors:** Wen Xu, Yijun Le, Jianzhong Zhang

**Affiliations:** ^1^ Department of Dermatology, Peking University People’s Hospital, Beijing, China; ^2^ Musculoskeletal Tumor Center, Peking University People’s Hospital, Beijing, China

**Keywords:** sebaceous gland carcinoma, overall survival rates, prognosis, risk factors, nomogram

## Abstract

**Background:**

Sebaceous gland carcinoma (SGC) is a rare tumor for which there are currently no effective tools to predict patient outcomes. We analyzed the clinical and pathological prognostic risk factors of sebaceous carcinoma based on population data and created a nomogram of related risk factors, which can more accurately predict the 3-, 5-, and 10-year overall survival (OS) rates of patients.

**Methods:**

SGC patients between 2004 and 2015 were collected from the Surveillance, Epidemiology, and End Results (SEER) database and randomly assigned to training and validation cohorts. Relevant risk factors were identified by univariate and multivariate COX hazards regression methods and combined to produce a correlation nomogram. The concordance index (C-index), the area under the receiver operating characteristic (AUC) curve, and calibration plots have demonstrated the predictive power of the nomogram. Decision curve analysis (DCA) was used to measure nomograms in clinical practice.

**Results:**

A total of 2844 eligible patients were randomly assigned to 70% of the training group (n=1990) and 30% of the validation group (n=854) in this study. The derived meaningful prognostic factors were applied to the establishment of the nomogram. The C-index for OS was 0.725 (95% CI: 0.706-0.741) in the training cohort and 0.710 (95% CI: 0.683-0.737) in the validation cohort. The AUC and calibration plots of 3-, 5-, and 10-year OS rates showed that the nomogram had good predictive power. DCA demonstrated that the nomogram constructed in this study could provide a clinical net benefit.

**Conclusion:**

We created a novel nomogram of prognostic factors for SGC, which more accurately and comprehensively predicted 3-, 5-, and 10-year OS in SGC patients. This can help clinicians identify high-risk patients as early as possible, carry out personalized treatment, follow-up, and monitoring, and improve the survival rate of SGC patients.

## Introduction

Sebaceous glands are holocrine glands widely distributed in the skin (1). Sebaceous gland carcinoma (SGC) accounts for approximately 0.2-4.6% of all skin cancers and is a rare and potentially aggressive tumor (2). SGC is often divided into periocular and extraocular sebaceous types. Extraocular SGC originates from the sebaceous glands, and periocular SGC is often thought to arise from the Meibomian and Zeiss glands of the eyelids (3). However, accounts of the origin of SGC are still inconsistent.

In the United States, the age-adjusted incidence rate for all SGC is 1-2.3 per million person-years, respectively, in a predominantly white population. Periocular type accounts for 26-27% of all SGC (4). Periocular type is most common in the upper eyelid, with a median age of 67.7 years and a majority of females. And extraocular type is more common in the head and neck, the median age of onset is 67.9 years old, and the majority is male (5). SGC usually appears as slow-growing yellowish, red nodules or plaques. Its clinical presentation mimics basal cell carcinoma (BCC), squamous cell carcinoma (SCC), or other sebaceous lesions (3). SGC is often misdiagnosed, so most sebaceous carcinomas are found to have peripheral invasion when the diagnosis is made. The mortality rate of SGC can be as high as 10% (6). Although the incidence of SGC is low, its mortality is also an issue that needs attention. Identifying the prognostic risk factors of SGC is of great significance for individualized treatment, follow-up, and management of patients.

In this study, we selected clinical and pathological characteristics related to SGC, such as age, gender, race, marital status, primary site, SEER stage, pathological grade (degree of tumor differentiation), and treatment regimen to evaluate the patient’s 3-, 5-, and 10-year overall survival rates, which were used to summarize prognostic risk factors for SGC. We created meaningful prognostic risk factors to establish a relevant nomogram, which can better guide follow-up treatment and monitoring for patients with SGC.

## Materials and methods

### Data source and selection of variables

The Surveillance, Epidemiology, and End Results (SEER) database is one of the largest cancer databases available to the public, covering approximately 28% of the US population. The clinical information, including sex, age, race, marital status, primary sites, SEER stage, grade, treatment, survival time, and survival status, were selected from the SEER research Plus Data,18 Regs, Nov 2020 Sub. The present research from the SEER program was conducted for all patients with diagnosed sebaceous carcinoma during2004-2015, The SEER research data were available using the SEER*Stat 8.4.0 (http://seer.cancer.gov//seerstat/). The exclusion criteria for this study were as follows: race unknown; no positive histology; primary site unknown; and treatment unknown. We eventually got 2844 patients based on the above inclusion and exclusion criteria. Patients were randomized into a training cohort(n=1990) and a validation cohort(n=854) in a 7:3 ratio. The SEER database agreement has been signed and provided permission to access SEER information (accession username: 12906-Nov2021). Since the SEER database is accessible to the public, we did not attempt institutional review board approval or informed consent for this study.

**Figure 1:**
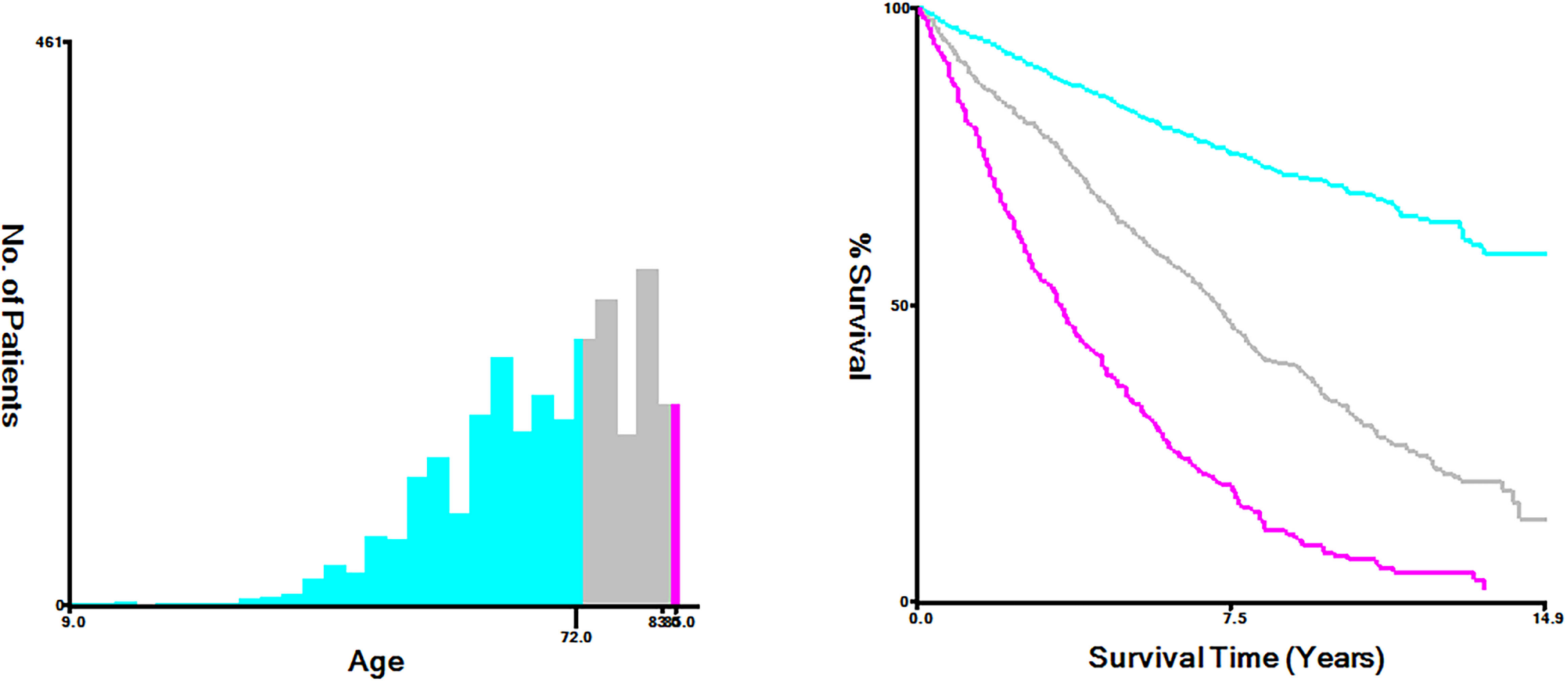
The optimal cut-off values for age were <72, 72-83, and >83 years old.

The optimal cutoff values were used to convert continuous variables into categorical variables with Xtile software (Yale University, New Haven, Connecticut, USA) (7). In this study, the optimal cutoff values by age were categorized into <72, 72-83 and >83years (**Figure 1**). The treatment included surgery, lymph node surgery (including lymph node dissection and sentinel lymph node biopsy), radiotherapy, and chemotherapy. Primary sites were classified into four sites as follows: head neck and face, trunk, extremity, and others (such as genital, mucous membrane, and overlapping area).

### Statistics analysis

All statistical analysis in our study was performed with R software version 4.1.3(https://www.r-project.org/). Descriptive statistics were used to analyze the demographic features of patients as well as clinical features. Using R software, the training and validation cohorts were randomly assigned, and the chi-square test was utilized to compare the associations between them. Univariate and multivariate cox regression analyses were performed to evaluate prognostic factors. Furthermore, we created a nomogram for predicting 3-, 5-, and 10-year OS using a multivariate Cox proportional hazard model. C-index, calibration curves (bootstrap 1,000 resampling validation), receiver operating characteristic (ROC) curves, and calculated areas under the receiver operating characteristic curve (AUC) values were used to evaluate the predictive capacity of the nomograms. The clinical value of the model was evaluated using decision curve analyses (DCAs).

## Results

### Demographic and clinicopathological characteristics

After screening, a total of 2844 patients diagnosed with SGC from 2004-2015 were finally obtained from the SEER database. The 2844 patients were divided into training cohort and validation cohort according to the ratio of 7:3. Demographically, the training and validation cohorts were predominantly young (49.2%), male (61.5%), Caucasian (86.0%), and married (40.2%). In terms of tumor characteristics, the head, face, and neck were the most common primary sites (71.2%). SEER stage was divided into local, regional, and distant, among which local staging was the most (75.7%). Pathological grades include grade I (highly differentiated), grade II (moderately differentiated), grade III (poorly differentiated), and grade IV (undifferentiated), among the known pathological grades, grade I/II is the main (20.0%). In terms of treatment, most patients received surgery (81.8%), and most patients did not receive lymph node surgery (94.4%). A small number of patients received adjuvant therapy, such as radiotherapy (4.0%) and chemotherapy (1.8%). More detailed information is in **Table 1**.

**Table 1:** The demographics and clinical features of patients with sebaceous gland carcinoma in different cohorts.

	Training group (N = 1990)	Validation group (N = 854)	Overall (N = 2844)	P-value
Age				
<73 years old	982 (49.3%)	416 (48.7%)	1398 (49.2%)	0.984
73-83 years old	632 (31.8%)	281 (32.9%)	913 (32.1%)	
>83 years old	376 (18.9%)	157 (18.4%)	533 (18.7%)	
Sex				
Female	735 (36.9%)	359 (42.0%)	1094 (38.5%)	0.037
Male	1255 (63.1%)	495 (58.0%)	1750 (61.5%)	
Race				
White	1704 (85.6%)	741 (86.8%)	2445 (86.0%)	0.894
Black	62 (3.1%)	21 (2.5%)	83 (2.9%)	
Other	224 (11.3%)	92 (10.8%)	316 (11.1%)	
Marital status				
Married	806 (40.5%)	338 (39.6%)	1144 (40.2%)	0.747
DSW	476 (23.9%)	225 (26.3%)	701 (24.6%)	
Unknown	708 (35.6%)	291 (34.1%)	999 (35.1%)	
Primary Site				
Head Neck and Face	1415 (71.1%)	609 (71.3%)	2024 (71.2%)	0.996
Trunk	332 (16.7%)	146 (17.1%)	478 (16.8%)	
Extremity	194 (9.7%)	76 (8.9%)	270 (9.5%)	
Other Sites	49 (2.5%)	23 (2.7%)	72 (2.5%)	
SEER Stage				
Localized	1505 (75.6%)	648 (75.9%)	2153 (75.7%)	1
Regional	78 (3.9%)	35 (4.1%)	113 (4.0%)	
Distant	100 (5.0%)	43 (5.0%)	143 (5.0%)	
Unstaged	307 (15.4%)	128 (15.0%)	435 (15.3%)	
Grade				
I/II	396 (19.9%)	173 (20.3%)	569 (20.0%)	0.998
III/IV	211 (10.6%)	87 (10.2%)	298 (10.5%)	
Unknown	1383 (69.5%)	594 (69.6%)	1977 (69.5%)	
Surgery				
No	365 (18.3%)	154 (18.0%)	519 (18.2%)	0.981
Yes	1625 (81.7%)	700 (82.0%)	2325 (81.8%)	
LN Surgery				
No	1889 (94.9%)	795 (93.1%)	2684 (94.4%)	0.151
Yes	101 (5.1%)	59 (6.9%)	160 (5.6%)	
Radiotherapy				
No	1917 (96.3%)	812 (95.1%)	2729 (96.0%)	0.3
Yes	73 (3.7%)	42 (4.9%)	115 (4.0%)	
Chemotherapy				
No	1956 (98.3%)	838 (98.1%)	2794 (98.2%)	0.954
Yes	34 (1.7%)	16 (1.9%)	50 (1.8%)	

DSW, Divorced Single Widowed; LN, Lymph Nodes.

### Selection of prognostic factors

**Figure 2:**
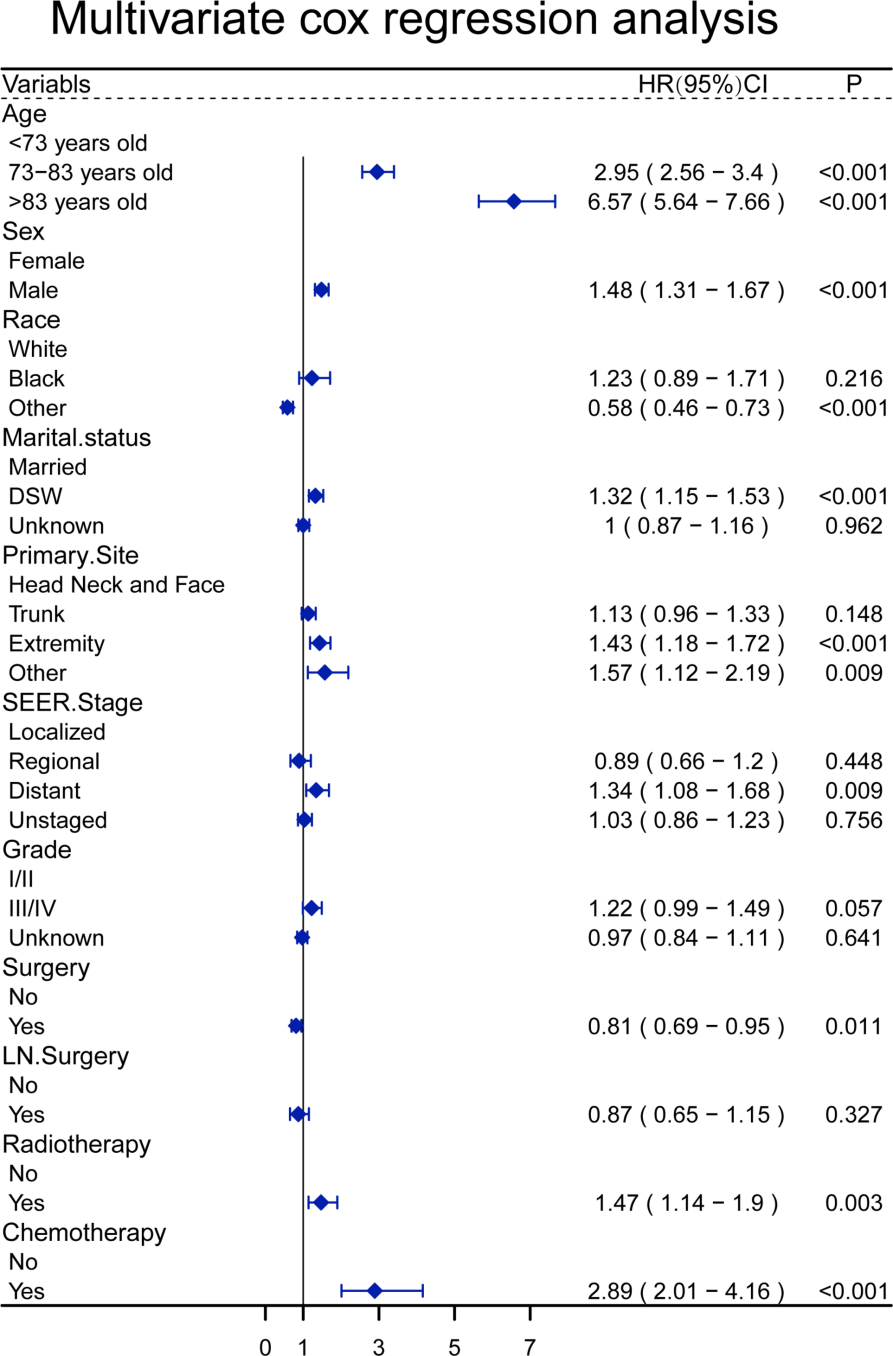
A forest plot to show the result of multivariate Cox regression analysis of patients with sebaceous gland carcinoma.

In a univariate analysis of OS, age (P<0.001), gender (P =0.024), race(P<0.001), marital status(P<0.001), primary site (P =0.012), SEER stage (P =0.001), pathological grade (P= 0.005), surgery(P <0.001), lymph node surgery(P =0.024), radiotherapy (P <0.001), chemotherapy(P <0.001) were related to OS (**Table 2**). The above variables were included in the multivariate analysis, and age, gender, race, marital status, primary site, SEER stage, surgical treatment, radiotherapy, and chemotherapy were associated with OS. The relative risk comparison can be seen in **Figure 2**.

**Table 2:** Univariable cox analysis for sebaceous gland carcinoma patients.

Characteristics	HR	95%CI	P
Age			
<73 years old	Reference		
73-83 years old	2.8	2.44-3.22	<0.001
>83 years old	6.3	5.45-7.29	<0.001
Sex			
Female	Reference		
Male	1.14	1.02-1.28	0.024
Race			
White	Reference		
Black	1.05	0.76-1.45	0.782
Other	0.52	0.42-0.65	<0.001
Marital status			
Married	Reference		
DSW	1.56	1.37-1.79	<0.001
Unknown	1.12	0.98-1.28	0.099
Primary Site			
Head Neck and Face	Reference		
Trunk	0.82	0.7-0.96	0.012
Extremity	1.15	0.95-1.38	0.144
Other	1.26	0.91-1.75	0.17
SEER Stage			
Localized	Reference		
Regional	1.22	0.93-1.22	0.001
Distant	1.36	1.10-1.69	0.005
Unstaged	0.99	0.86-1.14	0.862
Grade			
I/II	Reference	0.93-1.6	
III/IV	1.36	1.1-1.69	0.005
Unknown	1.16	0.99-1.35	0.068
Surgery			
No	Reference		
Yes	0.7	0.61-0.81	<0.001
LN Surgery			
No	Reference		
Yes	0.73	0.56-0.96	0.024
Radiotherapy			
No	Reference		
Yes	1.54	1.21-1.95	<0.001
Chemotherapy			
No	Reference		
Yes	2.74	1.96-3.84	<0.001

DSW, Divorced Single Widowed; LN, Lymph Nodes.

### Construction and validation of a nomogram

**Figure 3:**
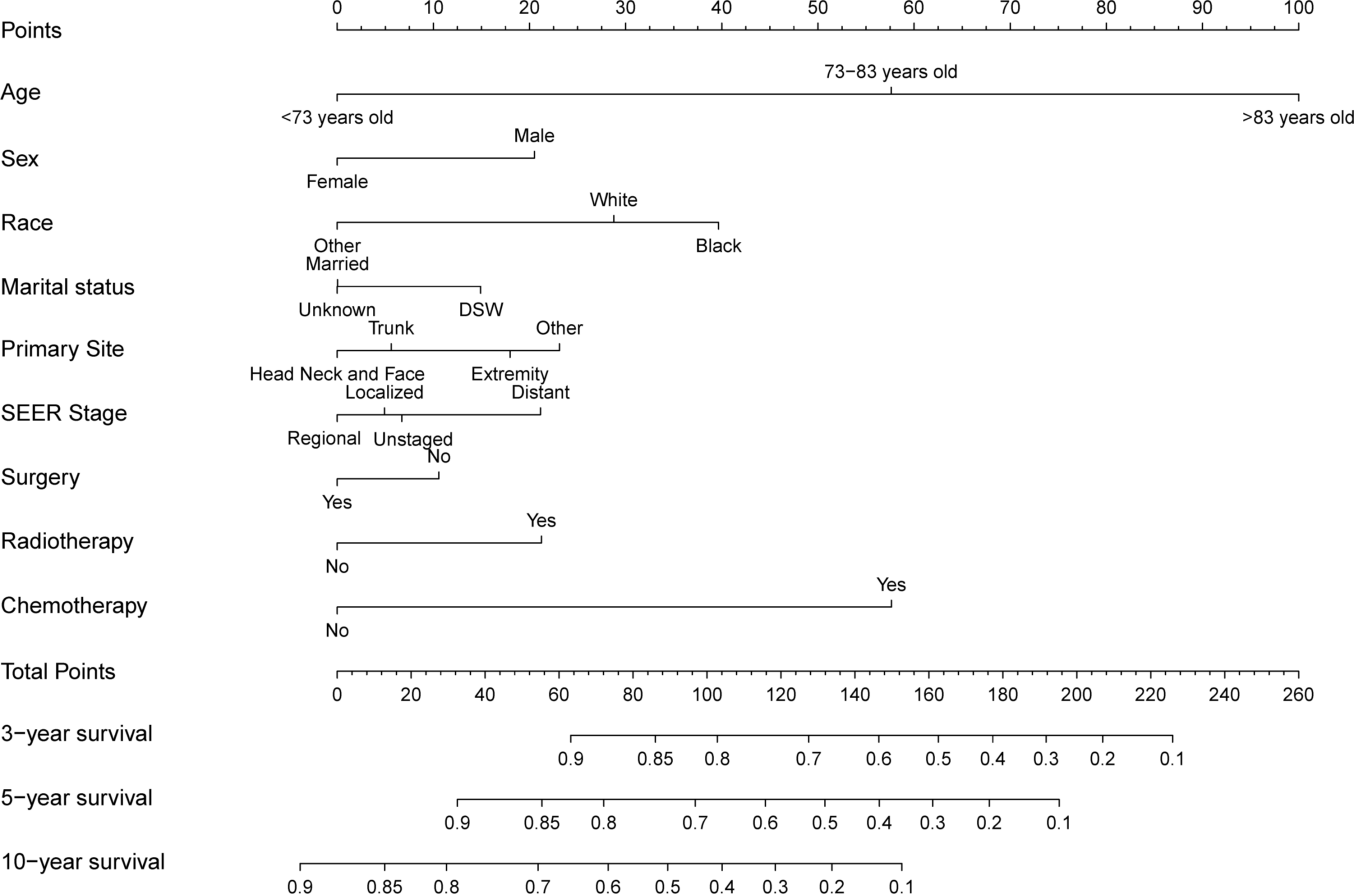
Nomogram for predicting 3-, 5- and 10-year overall survival rates of patients with sebaceous gland carcinoma.

**Figure 4:**
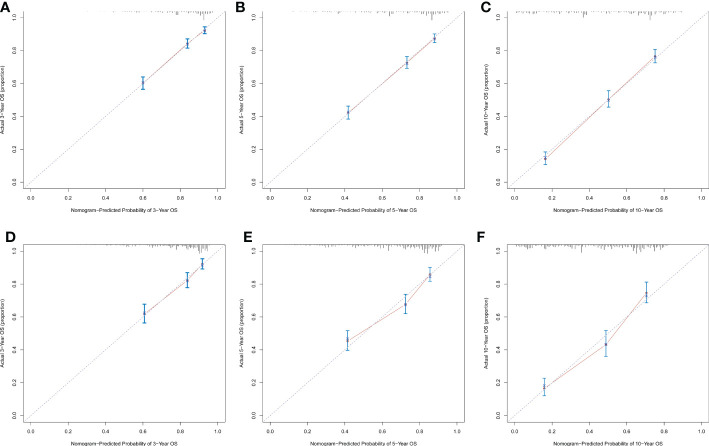
Calibration plots of the nomogram for predicting 3-, 5- and 10-year OS rates in sebaceous gland carcinoma patients. Calibration plots show the relationship between the predicted probabilities base on the nomogram and actual values of the training cohort **(A–C)** and validation cohort **(D–F)**.

**Figure 5:**
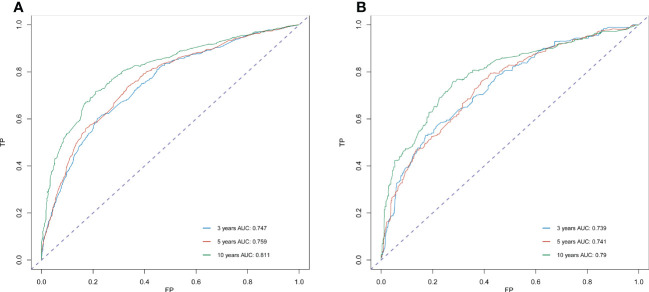
ROC curve analysis to predict 3-, 5- and 10-year OS rates in sebaceous gland carcinoma Patients. **(A)** ROC curve for the training cohort. **(B)** ROC curve for the external validation cohort. AUC, area under the curve; ROC, receiver operating characteristic; TP, true positive rates; FP, false positive rate.

**Figure 6:**
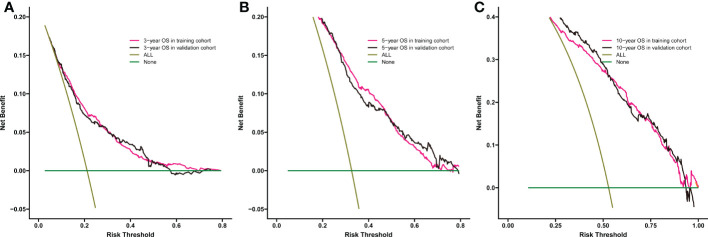
Comparison of the DCA curves of the nomogram for 3-year OS **(A)**,5-year OS **(B)**, and 10-year OS **(C)** prediction of the training cohort and validation cohort.

Significant independent risk factors from the multivariate analysis were used to construct the nomogram to predict 3-, 5-, and 10-year OS (**Figure 3**). The scale at the top of the nomogram provides a score for each prognostic variable, and the sum of all scores corresponds to the scale at the bottom of the nomogram for the nomogram display of OS prediction. The prognosis is mainly affected by age, followed by radiotherapy, chemotherapy, race, primary site, SEER stage, gender, marital status, and surgery. The nomogram was then validated by c-index, calibration curves (**Figure 4**), or ROC curves (**Figure 5**), and the DCA curves were used to evaluate clinical efficacy. The c-index for OS in the training cohort was 0.725 (95% CI: 0.706-0.741), while the c-index for OS in the validation cohort was 0.710 (95% CI: 0.683-0.737). The AUCs of 3-, 5-, and 10-year OS for the training cohort were 0.747, 0.759, and 0.811, respectively; meanwhile, the corresponding values for the validation cohort were 0.739, 0.741, and 0.790, respectively. All subsequent calibration curves showed satisfactory performance. The 3-, 5-, and 10-year DCA curves (**Figure 6**) showed that both models yield good benefits in both the training and validation cohorts.

## Discussion

In this study, based on univariate and multivariate cox proportional hazards regression analysis, we found that age, gender, race, marital status, primary site, SEER stage, surgery, radiotherapy, and chemotherapy were all prognostic risk factors for OS rate in SGC patients. At the same time, we created a nomogram to quantitatively predict the 3-, 5-, and 10-year OS rates of different individuals through the clinical and pathological characteristics of SGC. Through this nomogram, high-risk patients with SGC can be better identified, which is helpful for follow-up management and monitoring and can improve the survival rate of SGC patients.

Previous studies (8) have confirmed that advanced age affects the survival rate of SGC patients, and this was also verified in our study. SGC often occurs in the elderly, and the peak age of onset is about 67 years old (5). In our study, X-tile software was used to divide the age into three ranges of <72 years old, 72-83 years old, and >83 years old, which could better distinguish the effect of age on OS. Gender has not been identified as a risk factor for survival in previous studies. In this study, we found that the ratio of male to the female incidence of SGC was about 1.6:1. Both univariate and multivariate regression analyses demonstrated that gender could affect the prognosis of SGC, and males had a worse prognosis than females. We found that race was also a prognostic risk factor. It has been previously reported (9) that SGC is more common in Asians, and other study (10) have suggested that the incidence of SGC in yellow and white people is similar. The majority of the population included in this study were Caucasians, which was related to the limited statistical population in the SEER database. Our study showed that blacks have the worst prognosis, followed by whites, but the lack of a large amount of data from other races may lead to some bias in the research results. We still need larger populations and more comprehensive ethnic data to analyze the relationship between SGC and ethnicity. This study also evaluated the influence of marital status on the survival rate of SGC and showed that the survival rate of married patients was higher than that of single patients, which may be related to family care, disease emphasis, and psychological state.

It has been reported that the head and neck are the most common sites of SGC, with 74.2% of SGCs located outside the eye (4). The primary site has always been considered to be an important factor affecting prognosis. Clinical outcome analysis of 191 Asian-Indian patients with SGC confirmed that lacrimal canalicular invasion was associated with worse outcomes (11), and it has also been reported that SGC located in the lower eyelid or orbital extension has higher mortality than other sites (12). In this study, we found that the SGC of the head and neck had the best prognosis, followed by the trunk, and the extremities had the worse prognosis, while other sites (such as mucosa, etc.) had the worst prognosis. This was different from previous studies, considering that previous studies rarely analyzed SGC as a whole, but discussed it by category, which led to differences in the results of the studies. At the same time, consistent with the results of the previous study (11), the higher the SEER stage, the worse the prognosis. That is, surrounding tissue invasion, lymph node metastasis and distant metastasis were all poor prognostic factors for SGC. Previous study (13) reported that about 11-43.8% of extraocular SGCs were poorly differentiated. Poorly differentiated tumors have been reported (12, 14) to be associated with poor outcomes. However, in our study, the association between pathological grade and poor prognosis was not strong. We believe that it is related to the following reasons: firstly, the pathological grading of sebaceous gland carcinoma depends on many histological factors, such as site of involvement, multicenter origin, infiltrative growth pattern, vascular and lymphatic vessel invasion (15). Therefore, the precise pathological grading of sebaceous gland carcinoma is difficult. Secondly, pathological grading was not recorded in 1977 (69.5%) patients in the current study, which also posed some interference in our data analysis. In conclusion, more precise pathological grading is needed to further investigate its impact on the prognosis of SGC patients.

The first-line treatment for SGC is surgical resection. Treatment options should be individualized based on clinical presentation, medical history, and patient preference (5). This study also further confirmed that surgical resection can achieve a higher survival rate. There was a significant correlation between lymph node metastases at primary diagnosis and distant metastases and disease death. There was evidence (16) that SGC with lymph node metastases was more likely to spread to distant sites. Therefore, this study included lymph node surgery (including lymph node dissection and sentinel lymph node biopsy) in the analysis of prognostic factors, but we did not obtain meaningful results. Interestingly, we found that radiotherapy and chemotherapy were associated with poor outcomes. The use of radiotherapy for the treatment of SGC patients has been controversial. It has been reported (17) that radiotherapy may induce SGC. However, it has also been suggested that radiotherapy can be an alternative to surgical resection (18). Usually, the majority of patients treated with radiotherapy and chemotherapy are patients with metastatic SGC (5, 19), who have more severe disease, more rapid progression, and thus a worse prognosis. However, the toxic images during adjuvant radiotherapy and chemotherapy cannot be excluded. Radiotherapy and chemotherapy for SGC should be used with caution.

In this study, we included prognostic-related clinical and pathological characteristics, such as age, gender, race, marital status, primary site, SEER stage, pathological grade, and treatment method, through the large population data of the SEER database. These factors are readily available in the clinic and can better assess the risk of SGC patients. To the best of our knowledge, this study is the largest population-based study to date. In the present study, both the internal and external C-index was above 0.73, showing a pleasing discriminative ability to provide patients with prognostic information in a personalized manner. Likewise, AUC also implies good discriminative ability. The calibration curve shows that the predicted values of the nomogram have a high agreement. In addition, DCA was performed to provide the clinical net benefit of the predictive model. In this study, all results indicated that the DCA curves of the 3-, 5-, and 10-year OS rates of the new model yielded a significant net clinical benefit.

This study still had some limitations. Firstly, the population data provided by the SEER database comes from a portion of the U.S. population, which leads to racial limitations. As we mentioned earlier, there was a certain correlation between race and the incidence and survival of SGC. We need more complete ethnic data to complete the relevant research. Secondly, our prognostic risk factors were still insufficient. If the time of diagnosis (14), tumor size (11, 20), pagetoid spread (21), tumor growth pattern (20), immune marker tPD-1 (21)and other information can be combined into the nomogram, the prediction of the nomogram will be more accurate and more individual. Thirdly, other variables affecting survival were not controlled for in our study due to limited information registration in the database, which may lead to errors in the analysis. Finally, we had internal validation of the data, but external validation was lacking.

## Conclusion

In conclusion, we combined demographic and clinicopathological characteristics from the SEER database to build an efficient nomogram to predict prognostic factors in SGC patients. Among them, elder age, male, black race, unmarried, non-head, face and neck, lymph node or distant metastasis, no surgical resection, radiotherapy, and chemotherapy were all associated with poor outcomes. The nomogram we established can well combine relevant risk factors to predict the 3-, 5-, and 10-year OS rates of SGC patients. For patients with high prognostic risk factors, it is recommended to shorten the follow-up interval, and timely pay attention to whether recurrence, lymph node metastasis, and distant metastasis occur, which is of great significance for improving the prognosis of patients. We can use the nomogram to score patients’ prognostic risk values, provide patients with personalized treatment, monitor, and follow-up.

## Data availability statement

The raw data supporting the conclusions of this article will be made available by the authors, without undue reservation.

## Ethics statement

This study was undertaken without institutional review board approval or informed consent since the SEER database is publicly accessible.

## Author contributions

WX and JZ designed the study. YL was in charge of data collection and processing. The manuscript was written by WX and YL and was evaluated and modified by JZ. All authors contributed to the article and approved the submitted version.
